# Supplementary material for: Content-rich biological network constructed by mining PubMed abstracts
Source: BMC Bioinformatics. 2004 Oct 8;5:147. doi: 10.1186/1471-2105-5-147 (PMC528731; doi:10.1186/1471-2105-5-147)
Supplement: Additional File 2 — The original results of the above study (non-essential files are deleted to keep the file size under the limit set by BMC bioinformatics). [file 1471-2105-5-147-S2.bz2 › chilibotAdditionalFile2/dip10/640ID7939681E11027/html/FCCB_FCCA.html]

 


 **FCCB** and **FCCA** 
  
Found 9 abstracts in PubMed, retrieved 9.  
 

 What does Google say? 
 PDF only 
| .edu only 

---

**Interactive relationship** (e.g. stimulation, inhibition, etc)

**Inhibitory relationship**- The results suggest that the reduction of methacrylate or acrylate by benzyl viologen radical is most likely catalyzed either by the periplasmic flavoprotein  **FccA**  or by a complex consisting of  **FccA**  and the predicted c type cytochrome  **FccB** .  Ref: 11685377 Arch Microbiol, 2001
**Stimulatory relationship**- All cultures that contained sulfide in the medium had elevated levels of FCSD  [ **FCCA** ]   [ **FCCB** ]  RNA compared with cells grown on organics acetate, malate, or succinate or thiosulfate alone, consistent with the role of FCSD  [ **FCCA** ]   [ **FCCB** ]  in sulfide oxidation.  Ref: 10809687 J Bacteriol, 2000
**Neutral relationship**- As indicated by Northern blot analysis,  **fccA**  is cotranscribed with  **fccB**  and fccC.  Ref: 9560424 Arch Microbiol, 1998
- The membrane localization of FCSD  [ **FCCA** ]   [ **FCCB** ]  was confirmed by Western blotting with antibodies raised against Chromatium vinosum FCSD  [ **FCCA** ]   [ **FCCB** ] .  Ref: 10809687 J Bacteriol, 2000

**Non-interactive relationship** (e.g. studied together, co-existance, homology, etc.)

- 4 We have established that the constitutively expressed FCSD  [ **FCCA** ]   [ **FCCB** ]  genes soxEF1 are located elsewhere in the genome.  Ref: 11863431 Biochemistry, 2002
- by the Family and Child Services Department FCSD  [ **FCCA** ]   [ **FCCB** ] .  Ref: 12041067 Dom Law Rep< MedlineTA>Dom Law Rep, 2002
- In order to protect the unborn baby s health, the Superintendent of the FCSD  [ **FCCA** ]   [ **FCCB** ]  ordered its apprehension.  Ref: 12041067 Dom Law Rep< MedlineTA>Dom Law Rep, 2002
- The genetic context of FCSD  [ **FCCA** ]   [ **FCCB** ]  is different from that in other species in that flanking genes are not conserved.  Ref: 10809687 J Bacteriol, 2000
- The transcript is only large enough to encode the two FCSD  [ **FCCA** ]   [ **FCCB** ]  subunits.  Ref: 10809687 J Bacteriol, 2000
- Efforts to produce the holoproteins of the soluble Allochromatium vinosum FCSD  [ **FCCA** ]   [ **FCCB** ]  and the membrane bound Ectothiorhodospira vacuolata protein in Escherichia coli using several expression systems were unsuccessful.  Ref: 11479699 Arch Microbiol, 2001
- SoxF was 37% identical to the flavoprotein  **FccB**  of flavocytochrome c  [ **FCCA** ]  sulfide dehydrogenase of Allochromatium vinosum.  Ref: 11443084 J Bacteriol, 2001
- The flavoprotein subunit of FCSD  [ **FCCA** ]   [ **FCCB** ]  is most closely related to glutathione reductase GR in three dimensional structure and, like that protein, contains three domains.  Ref: 8880899 Protein Sci, 1996
- These data allowed isolation of a 5.5 kb subgenomic clone which contains the cytochrome gene and an adjacent flavoprotein gene as in other species which have an FCSD  [ **FCCA** ]   [ **FCCB** ] .  Ref: 10809687 J Bacteriol, 2000
- FCSD  [ **FCCA** ]   [ **FCCB** ]  is a heterodimer containing two different cofactors, a flavin FAD and one or two heme c groups, located on the separate protein subunits.  Ref: 11479699 Arch Microbiol, 2001
- When aligned according to the three dimensional structure of Chromatium FCSD  [ **FCCA** ]   [ **FCCB** ] , all but one of the side chains near the flavin are conserved.  Ref: 10809687 J Bacteriol, 2000
- The amino acid sequence of the flavoprotein subunit of Chromatium vinosum flavocytochrome c  [ **FCCA** ]  sulfide dehydrogenase FCSD  [ **FCCA** ]   [ **FCCB** ]  was determined by automated Edman degradation and mass spectrometry in conjunction with the three dimensional structure determination Chen Z et al.  Ref: 8880899 Protein Sci, 1996
- Furthermore, Northern hybridization showed that the production of E. vacuolata FCSD  [ **FCCA** ]   [ **FCCB** ]  mRNA is regulated by sulfide.  Ref: 10809687 J Bacteriol, 2000
- thiosulfatophilum flavocytochrome c  [ **FCCA** ]  sulfide dehydrogenase FCSD  [ **FCCA** ]   [ **FCCB** ] , having 49% identity.  Ref: 10809687 J Bacteriol, 2000
- Domains 1 and 2 of FCSD  [ **FCCA** ]   [ **FCCB** ] , as of GR, are homologous to one another and represent an ancient gene doubling.  Ref: 8880899 Protein Sci, 1996
- The Supreme Court found that a fetus was not subject to apprehension within the meaning of the legislation under which the FCSD  [ **FCCA** ]   [ **FCCB** ]  acted.  Ref: 12041067 Dom Law Rep< MedlineTA>Dom Law Rep, 1996
- Flavocytochrome c  [ **FCCA** ]  sulfide dehydrogenase FCSD  [ **FCCA** ]   [ **FCCB** ] , an enzyme that catalyzes the reversible conversion of sulfide to elemental sulfur in vitro, is common to bacteria that utilize reduced sulfur compounds as electron donors in the process of carbon dioxide fixation.  Ref: 11479699 Arch Microbiol, 2001
- Domain 2 of GR is the site of NADP binding, but has an unknown role in FCSD  [ **FCCA** ]   [ **FCCB** ] .  Ref: 8880899 Protein Sci, 1996
- b medium with dextran coated charcoal treated growth factor inactivated serum DCC FCSd  [ **FCCA** ]   [ **FCCB** ] .  Ref: 2268550 J Steroid Biochem Mol Biol, 1990
- Both Rhodobacter hosts were able to transcribe the genes coding for FCSD  [ **FCCA** ]   [ **FCCB** ]  from the rbcA promoter and to produce detectable amounts of recombinant FCSD  [ **FCCA** ]   [ **FCCB** ]  holoprotein.  Ref: 11479699 Arch Microbiol, 2001
- The third domain provides the dimerization interface for GR, but is the site of binding of the cytochrome subunit in FCSD  [ **FCCA** ]   [ **FCCB** ] .  Ref: 8880899 Protein Sci, 1996
- However, soluble cytochrome c 551 and flavocytochrome c  [ **FCCA** ]  FCSD  [ **FCCA** ]   [ **FCCB** ]  have previously been implicated in the oxidation of thiosulfate and sulfide on the basis of enzyme assays in Chlorobium.  Ref: 11863431 Biochemistry, 2002
